# Supplementary material for: Characterizing the limitations of using diagnosis codes in the context of machine learning for healthcare
Source: BMC Med Inform Decis Mak. 2024 Feb 14;24:51. doi: 10.1186/s12911-024-02449-8 (PMC10868117; doi:10.1186/s12911-024-02449-8)
Supplement: Supplementary file 1 — Supplementary Material 1 [file 12911_2024_2449_MOESM1_ESM.docx]

**Appendix 1. Thresholds for each severity level of the lab-based labels**

| Outcome | Test | Unit | Mild | Moderate | Severe |
| --- | --- | --- | --- | --- | --- |
| Acute Kidney Injury | Creatinine | µmol/L | ≥1.5x baseline^1^ or increase of 26.5 | ≥2x baseline | ≥3x or increase of 353.6 |
| Hyperkalemia | Potassium | mmol/L | >5.5 | >6 | >7 |
| Hypoglycemia | Glucose | mmol/L | <3.9 | <3.5 | <3 |
| Hyponatremia | Sodium | mmol/L | ≤135 | <130 | <125 |
| Neutropenia | Absolute neutrophil count^2^ | 10^9^/L | <1.5 | <1.0 | <0.5 |
| Anemia | Hemoglobin | g/L | <120 | <110 | <70 |
| Thrombocytopenia | Platelet | 10^9^/L | <150 | <100 | <50 |

^1^ Baseline defined as the minimum creatinine over the 3-month period prior to the index time, or the upper bound of the normal reference range in the absence of an observed value

^2^ Absolute neutrophil count was the sum of neutrophil and band counts. If a complete blood count differential was not performed or not reported, we categorized neutropenia if the total white blood cell count met the threshold

**Appendix 2. SNOMED codes used to define diagnosis-based labels for StanfordPeds and StanfordAdults***

| OMOP  Concept ID | OMOP  Concept Name | SNOMED  Code |
| --- | --- | --- |
| Acute Kidney Injury |  |  |
| 444044 | Acute tubular necrosis | 35455006 |
| 197320 | Acute renal failure syndrome | 14669001 |
| 432961 | Acute renal papillary necrosis with renal failure | 298015003 |
| Hyperkalemia |  |  |
| 434610 | Hyperkalemia | 14140009 |
| Hypoglycemia |  |  |
| 4226798 | Hypoglycemic coma due to diabetes mellitus | 421725003 |
| 45769876 | Hypoglycemia due to type 1 diabetes mellitus | 84371000119108 |
| 380688 | Hypoglycemic coma | 267384006 |
| 4048805 | Non-diabetic hypoglycemic coma | 230796005 |
| 36714116 | Hypoglycemic coma due to type 2 diabetes mellitus | 719216001 |
| 4096804 | Drug-induced hypoglycemia without coma | 190448007 |
| 24609 | Hypoglycemia | 302866003 |
| 4029423 | Hypoglycemia due to diabetes mellitus | 237633009 |
| 4029424 | Non-diabetic hypoglycemia | 237637005 |
| 45757363 | Hypoglycemia due to type 2 diabetes mellitus | 120731000119103 |
| 23034 | Neonatal hypoglycemia | 52767006 |
| 4228112 | Hypoglycemic coma due to type 1 diabetes mellitus | 421437000 |
| Hyponatremia |  |  |
| 4232311 | Hyponatremia | 89627008 |
| Neutropenia |  |  |
| 320073 | Neutropenia | 165517008 |
| Anemia |  |  |
| 37398911 | Anemia in chronic kidney disease stage 4 | 691401000119104 |
| 35624756 | Anemia due to and following chemotherapy | 767657005 |
| 439777 | Anemia | 271737000 |
| 4006467 | Anemia due to infection | 111570005 |
| 37017132 | Anemia co-occurrent with human immunodeficiency virus infection | 713349004 |
| 37018722 | Anemia caused by zidovudine | 713496008 |
| 37395652 | Anemia in chronic kidney disease stage 5 | 691411000119101 |
| Thrombocytopenia |  |  |
| 432870 | Thrombocytopenic disorder | 302215000 |

***** For each SNOMED concept we additionally included its descendants, that is, any concept that has an “is a” relationship with the listed concept.

Abbreviation: SNOMED: Systematized Nomenclature of Medicine Clinical Terms; Peds: pediatrics;

**Appendix 3. ICD10 codes used to define diagnosis-based labels in SickKids.**

| **ICD Code** | **Description** |
| --- | --- |
| Acute Kidney Injury |  |
| N17.0 | Acute renal failure with tubular necrosis |
| N17.8 | Other acute renal failure |
| N17.9 | Acute renal failure, unspecified |
| Hyperkalemia |  |
| E87.5 | Hyperkalemia |
| P74.31 | Hyperkalemia of newborn |
| Hypoglycemia |  |
| E16.0 | Drug-induced hypoglycaemia without coma |
| E16.1 | Other hypoglycaemia |
| E16.2 | Hypoglycaemia, unspecified |
| P70.4 | Other neonatal hypoglycaemia |
| Hyponatremia |  |
| E87.1 | Hypo-osmolality and hyponatraemia |
| P74.20 | Hypernatremia of newborn |
| E86.0 | Dehydration (with hyponatremia) |
| Neutropenia |  |
| D70.0 | Congenital agranulocytosis |
| Anemia |  |
| D50.0 | Iron deficiency anemia secondary to blood loss (chronic) |
| D50.8 | Other iron deficiency anemias |
| D50.9 | Iron deficiency anemia, unspecified |
| D51.0 | Vitamin B12 deficiency anemia due to intrinsic factor deficiency |
| D51.3 | Dietary folate deficiency anemia |
| D51.9 | Vitamin B12 deficiency anemia, unspecified |
| D52.0 | Dietary folate deficiency anemia |
| D52.9 | Folate deficiency anemia, unspecified |
| D53.2 | Scorbutic anemia |
| D55.0 | Anemia due to glucose-6-phosphate dehydrogenase [G6PD] deficiency |
| D55.2 | Anemia due to disorders of glycolytic enzymes |
| D55.9 | Anemia due to enzyme disorder, unspecified |
| D57.1 | Sickle-cell anemia without crisis |
| D58.0 | Hereditary spherocytosis |
| D59.1 | Autoimmune hemolytic anemia |
| D59.4 | Other nonautoimmune hemolytic anemias |
| D59.6 | Hemoglobinuria due to hemolysis from other external causes |
| D59.9 | Acquired hemolytic anemia, unspecified |
| D60.9 | Acquired pure red cell aplasia, unspecified |
| D61.9 | Aplastic anemia, unspecified |
| D62 | Acute posthemorrhagic anemia |
| D63.8 | Anemia in chronic diseases classified elsewhere |
| D64.3 | Other sideroblastic anemias |
| D64.8 | Other specified anemias |
| D64.9 | Anemia, unspecified |
| D69.6 | Anemia with low platelet count |
| O99.003 | Anemia complicating pregnancy, third trimester |
| P61.2 | Neonatal anemia due to acute blood loss |
| P61.4 | Neonatal anemia due to delayed fetal to neonatal transfusion |
| Thrombocytopenia |  |
| D18.1 | Hemangioma, any site |
| D69.1 | Qualitative platelet defects |
| D69.38 | Other primary thrombocytopenia |
| D69.4 | Other primary thrombocytopenia |
| D69.5 | Secondary thrombocytopenia |
| D69.6 | Thrombocytopenia, unspecified |
| P61.0 | Transient neonatal thrombocytopenia |
| Q87.2 | TAR (thrombocytopenia with absent radius syndrome) |

Abbreviation: ICD10: international classification of diseases 10^th^ edition; SickKids: The Hospital for Sick Children.

**Appendix 4. Institution- and age group-specific threshold for abnormal lab test for SickKids (blue) and StanfordPeds (red)**


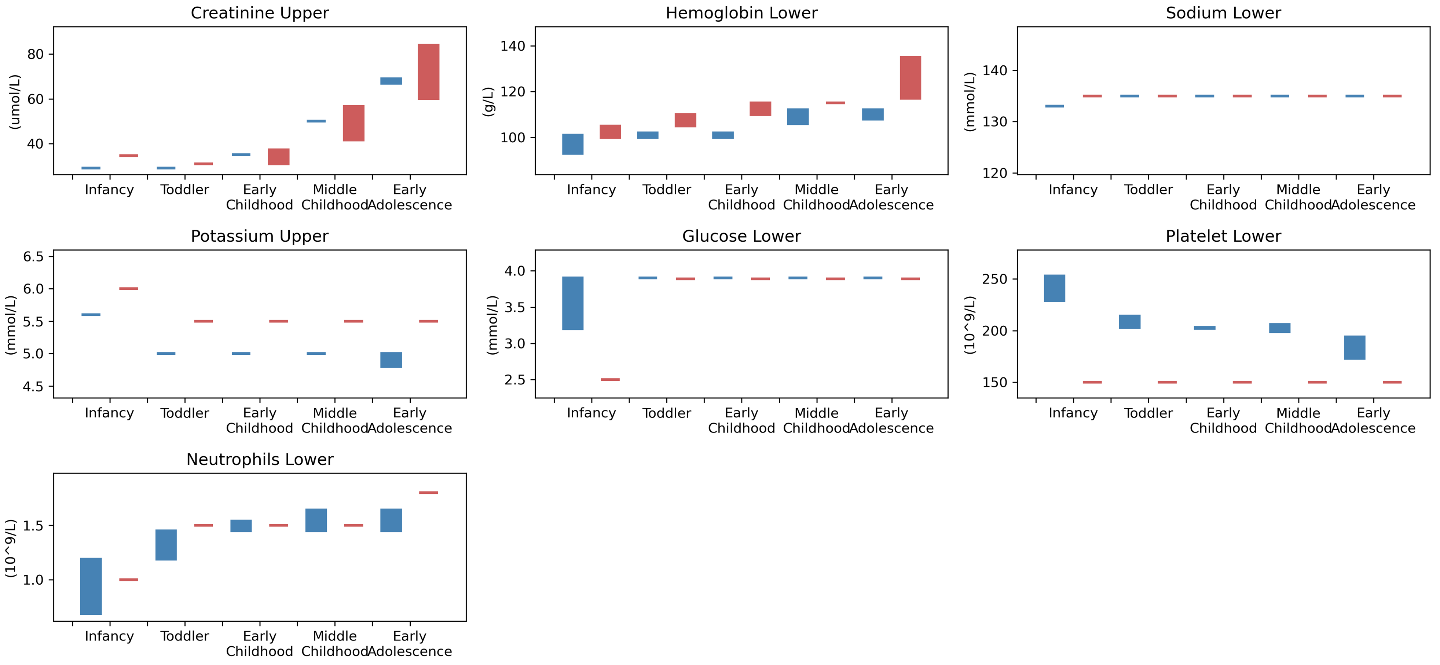


Abbreviation: SickKids: The Hospital for Sick Children; Peds: pediatrics.

**Appendix 5. Average number of lab tests per inpatient day across all admissions by cohort**

|  | SickKids  Mean±SD | StanfordPeds  Mean±SD | StanfordAdults  Mean±SD | P Value*  SickKids vs. StanfordPeds |
| --- | --- | --- | --- | --- |
| Creatinine | 0.19±0.33 | 0.36±0.45 | 0.70±0.54 | <0.001 |
| Potassium | 0.39±0.82 | 0.62±1.09 | 0.76±0.69 | <0.001 |
| Glucose | 0.45±1.39 | 0.70±1.42 | 1.37±1.94 | <0.001 |
| Sodium | 0.38±0.81 | 0.72±1.27 | 0.88±0.92 | <0.001 |
| Neutrophils | 0.15±0.26 | 0.28±0.46 | 0.72±0.71 | <0.001 |
| Hemoglobin | 0.22±0.34 | 0.53±1.02 | 0.76±0.55 | <0.001 |
| Platelet | 0.22±0.34 | 0.23±0.35 | 0.71±0.49 | <0.001 |

* P value obtained using mixed-effects linear regressions with lab testing frequency as the outcome, institution and pediatric age group as fixed effects and subject as random intercept.

Abbreviation: SickKids: The Hospital for Sick Children; Peds: pediatrics; SD: standard deviation
